# A Case of Death from Umbilical Hemorrhage

**Published:** 1873-10

**Authors:** Norman Bridge

**Affiliations:** Chicago


### Article VI.—*A Case of Death from Umbilical Hemorrhage.*

Reported by Norman Bridge, M.D., Chicago.

The following notes are submitted, as adding one more case to the list of deaths from an unusual accident.

Mrs. F., aged about 42, in appearance large and robust, was delivered of her fourteenth child on January 3rd, 1873, after a labor of less than three hours. It was her belief that the time was a month short of full term. The child was small, but seemed healthy; it thrived until the 10th inst., the cord having shriveled and dropped off a day or two before.

I had not seen the case for several days, and was called hurriedly on the morning of the 10th, to find that the child had been bleeding from the umbilicus for six or more hours. All the bandages and clothing were saturated with blood, indicating the loss of several ounces. The child appeared thoroughly exsanguinated, and had a rapid and irregular pulse.

Powdered per-sulphate of iron was applied to the bleeding surface, and a thick compress bandaged tightly against it. There was ordered frequent doses of brandy.

No more bleeding occurred, but the child died during the afternoon.

The surface of the umbilicus was so much retracted that the particular vessels yielding the blood were not made out.

